# *‘We got more than we expected.’* Older people’s experiences of falls-prevention exercise interventions and implications for practice; a qualitative study

**DOI:** 10.1017/S1463423619000379

**Published:** 2019-07-01

**Authors:** Natasher Lafond, Asiya Maula, Steve Iliffe, Kavita Vedhara, Sarah Audsley, Denise Kendrick, Elizabeth Orton

**Affiliations:** 1 Research Fellow, University of Nottingham, School of Medicine, United Kingdom; 2 ACF GPR, University of Nottingham, School of Medicine, United Kingdom; 3 Professor of Primary Care for Older People (Now Emeritus Professor), University College London, Research Department of Primary Care & Population Health, United Kingdom; 4 Professor of Health Psychology, University of Nottingham, School of Medicine, United Kingdom; 5 Posdoctoral fellow, University of Nottingham, School of Medicine, United Kingdom; 6 Professor of Primary Care Research, University of Nottingham, School of Medicine, United Kingdom; 7 Associate Professor and Consultant in Public Health, University of Nottingham, School of Medicine, United Kingdom

**Keywords:** older people, physical activity, barriers and facilitators, falls-prevention

## Abstract

**Aim:**

To explore the experiences of older adults participating in strength and balance exercise programmes and understand participants’ rationale for programme uptake and completion.

**Background:**

Regular physical activity, specifically strength and balance exercises, has been shown to improve health and well-being and reduce the risk of falling in older adults. With the number of people living into older age increasing, understanding older people’s experiences of strength and balance programmes and what encourages their take-up and completion is extremely important. This paper reports on the qualitative experiences of older adults that previously participated in ProAct65+, a randomised controlled trial of Falls Management Exercise (FaME) programme and Otago Exercise Programme (OEP) versus usual care.

**Methods:**

Ten general practices in Nottinghamshire and Derbyshire, England, who participated in the ProAct65+ trial were approached to take part. Using maximum variation sampling (age, gender, falls history, fear of falling and trial arm) we recruited, via the practices, 30 people that had participated in the FaME (*n* = 15) or OEP (*n* = 15) trial arms. Participants were interviewed in their own homes. Interviews were audio-recorded, transcribed verbatim and thematically analysed.

**Findings:**

We identified five themes: choice of exercise programme; commitment, discipline and motivation; benchmarking, feedback and monitoring; benefits of the exercise programmes and reactions to the end of the programmes. There were four sub-themes within the benefits theme: pleasure and boredom, social interaction and isolation, physical benefits, and knowledge and understanding.

This study has outlined the experiences and identified specific barriers and facilitators to uptake and completion of falls-prevention exercises by older adults. The perspective and experiences of these participants is important if programmes are to be designed to meet the needs of the target population. Insights from this study will enable commissioners to develop and provide appropriate falls-prevention exercise programmes that encourage high uptake and programme completion.

## Introduction

In the United Kingdom, the number of people living into older age is increasing, with over 11.4 million people (18% of the population) aged 65 and over in mid-2016 (Office for National Statistics). Older people are the most sedentary group in the United Kingdom. Only 57% of men and 52% of women aged 65–74 years and 43% of men and 21% of women aged 75–84 years meet the recommended physical activity guidelines for aerobic activity (at least 150 min per week of moderately intensive physical activity or 75 min per week of vigorous physical activity) (Scholes and Mindell, 2012).

Regular physical activity improves health and well-being (Department of Health, 2009) and reduces the risk of type 2 diabetes, osteoporosis, cardiovascular disease and some cancers (Bauman, 2004, Department of Health, 2005) with potential savings for the National Health Service (NHS Right Care) (Blair *et al*., 1996, Roux *et al*., 2008, World Health Organization, 2010). In addition to aerobic activity, physical activity that improves muscle strength and balance is particularly important for older people in order to prevent falls, and falls prevention in older adults is important given that each year 28–35% of people aged 65 years and over fall (World Health Organization, 2010). Strength and balance training is effective in reducing the risk of falls in older people living in the community (Gillespie *et al*., 2012) but only if it is progressively difficult, tailored to the individual’s functional ability and is of sufficient ‘dose’ (minimum 50 h in total over 6 months) (Sherrington *et al*., 2008). Understanding the factors that improve programme uptake and completion, thus achieving this minimum dose, is therefore of critical importance.

We recently demonstrated that a community-based strength and balance exercise programme was effective in increasing physical activity and reducing falls in older adults (The ProAct 65+ trial) (Iliffe *et al*., 2010, 2014), but we did not explore participants’ views of the programmes at this time. The ProAct65+ trial was a pragmatic, three-arm parallel design, cluster randomised controlled trial (cRCT). The three arms were a community-based group exercise programme – Falls Management Exercise (FaME) programme (Skelton and Dinan, 1999, Skelton *et al*., 2005); Otago Exercise Programme (OEP) – a home-based exercise programme (Campbell *et al*., 1997, Gardner *et al*., 2001, Liu-Ambrose *et al*., 2008) and usual care. In the current study, we revisited a sample of ProAct65+ participants from the intervention arms and explored qualitatively the factors that led to their initial uptake and then ongoing programme attendance.

## Methods

We undertook a qualitative study exploring older people’s experiences of two evidence-based exercise programmes designed for older people and studied in the ProAct65+ cRCT.

### Exercise programmes

The FaME programme was a weekly hour-long postural stability group exercise class delivered in local community settings, supplemented with two 30-min home exercise sessions per week (based on the OEP, with an instruction booklet) for 24 weeks. Participants were also advised to walk at least twice per week for up to 30 min. The OEP consisted of 30 min of progressively difficult strength and balance exercises to be performed at home at least three times per week, supplemented with an exercise diary and walking plan for up to 30 min at a moderate pace to be undertaken at least two times per week for 24 weeks (Iliffe *et al*., 2010, 2014).

### Selection of participants

The 10 general practices in Nottinghamshire and Derbyshire which recruited the largest number of participants for the ProAct 65+ trial were approached to take part in this study. The first five practices that agreed to participate were included. Practices were given a list of their patients who took part in the ProAct 65+ trial and were asked to check if participants were still alive, still registered at the practice and met the eligibility criteria. Participants were eligible to participate if they were able to give informed consent, did not have a terminal illness, were English speakers and if the GP thought that inviting them to the study would not cause undue distress. GPs sent the study invites to those eligible and one reminder to non-responders three weeks later.

Those expressing an interest in taking part in the study returned reply slips to the research team. Maximum variation sampling using baseline data from the ProAct 65+ trial [age, gender, falls history, fear of falling and trial arm (FaME or OEP)] was used to ensure diversity across these characteristics where possible. Those sampled were telephoned by the researcher to explain the study and answer any questions. Interviews were arranged with those agreeing to participate and consent forms signed prior to interviews. We aimed to recruit 30 participants.

### Qualitative interview

One-to-one semi-structured face-to-face interviews were conducted at participants’ homes. Semi-structured interviews were used as they allow in-depth exploration of participants’ responses (Galletta and Cross, 2013) and because older people may not wish to discuss their exercise habits and preferences in a group environment. The first two interviews served as pilot interviews to test the interview schedule content and structure. As a result of the pilot interviews, the order in which the questions were asked by the interviewer was modified in order to improve the flow of the interview. Two researchers (NL and AM) conducted interviews, which lasted between 12 min and 44 min. To ensure consistency of interview technique, interview shadowing was employed. One researcher conducted the interview and the second observed, followed by a debrief between the interviewers. This process was repeated with roles reversed until both interviewers agreed that their interview techniques were consistent.

Interviews explored participants’ expectations, experiences and thoughts about exercise, their views about being assigned to either the FaME or OEP arm of the ProAct65+ trial and their feelings at the end of the FaME or OEP programmes.

### Data analysis

Interview data were transcribed verbatim. Data were managed using NVivo 10 (QSR international) and analysis was undertaken using the Framework Analysis approach (Green and Thorogood 2009).

One researcher (NL) coded 30 interview transcripts and the second (AM) coded 19 transcripts. At an early stage during the analysis, three randomly selected interviews were coded independently by the two researchers (NL and AM) to check for consistency and to raise coding queries. The initial framework was developed using the preliminary analysis, the existing literature and utilising team expertise. Further analysis of the complete dataset (30 participant interviews) continued in NVivo 10, the initial themes were developed and expanded by the two researchers (NL and AM). A patient and public involvement (PPI) member helped with the qualitative data analysis by reviewing six transcripts and the coding framework to ensure the patient perspective was incorporated into the analysis. The coding framework was discussed with the wider research team (DK, EO, SI). At each stage of the analysis, emerging themes and discrepancies were discussed between NL and AM. Once the coding was complete, NL and AM reviewed each other’s coding on five further interviews and coding alterations made as necessary across all 30 interviews.

## Results

The five practices had consented a total of 122 older people for the ProAct 65+ trial. After eligibility checks were carried out, 99 of the 122 ProAct 65+ trial participants were invited to take part in this study. A total of 53 participants returned expressions of interest and 30 were interviewed between December 2015 and March 2016 (see Table 1 for participant characteristics, Keeping Active baseline data). Fifteen participants had been in the OEP home exercise group; 12 female and 3 male participants aged between 70 and 95 years. The remaining 15 participants were from the FaME group; 10 female and 5 male participants aged between 71 and 88 years.

**Table 1.** Keeping active participant characteristics: baseline data

	**Exercise group**	
	**FaME**	**OEP**
**Patient characteristics**		
Female sex, *n* (%)	10 (66.7)	12 (80.0)
Age, years mean [SD]	76.47 [5.6]	78.47 [5.7]
Household, *n* (%)		
Lives alone	5 (33.3)	6 (40.0)
Lives with other family members	0 (0.0)	2 (13.3)
Married or living as married	10 (66.7)	7 (46.7)
Employment, *n* (%)		
In part-time employment	1 (6.7)	0 (0.0)
Homemaker	0 (0.0)	1 (6.7)
Retired	14 (93.3)	14 (93.3)
Minutes of moderate-to-vigorous physical activity per week^a^ mean	173 (130.5)	177 (148.8)
Current level of activity, *n* (%)		
I do not do any planned physical activity during the week and would find it difficult to start	0 (0.0)	3 (20.0)
I used to exercise regularly each week but have lapsed	2 (13.3)	0 (0.0)
I exercise once in a while but not weekly	0 (0.0)	4 (26.7)
I exercise regularly each week	13 (86.7)	8 (53.3)
Falls risk^b^ median (IQR)	1.00 (0–1)	1.00 (0–1)

SD = standard deviation; IQR = interquartile range

a Community Healthy Activities Model Programme for Seniors (CHAMPS) (Mathews *et al*.) Data from Keeping Active participants

b Falls Risk Assessment Tool (FRAT; range 0–5)

Five key themes emerged from the data (see also Table 2). These were (1) choice in the type of exercise programme; (2) commitment, discipline and motivation; (3) benchmarking, feedback and monitoring; (4) benefits of the exercise programmes and (5) reactions to the end of the FaME or OEP programmes. There were four sub-themes within the benefits theme: (a) pleasure and boredom; (b) social interaction and isolation; (c) physical benefits and (d) knowledge and understanding.

**Table 2.** Themes, sub-themes and supplemental illustrative data extracts

Theme	Sub-theme	Illustrative data extracts
Choice in the type of exercise programme		*I was a bit disappointed I wasn’t in the classes group to be honest and I think that would have been good to do it with other people, I always like things where you are helping each other along the way and moaning together and everything so yes I think I was a bit disappointed that it was to do at home but as it worked out I think it was fine to do at home*. (P01 Female 70 OEP)
Commitment, discipline and motivation		*The teacher was good and she was… there is no other word, I think she pushed us and we erm responded effectively*. (P18 Male 78 FaME) *Just simply that I didn’t do it as much as I should have done with being on my own… it is a help if you have got the willingness to do it and you have got to really push yourself to do it*. (P30 Female 77 OEP)
Benchmarking, feedback and monitoring		*When you are at home you have got no stimulation more or less when you’re at home but when you are in a class you try and do the same as everybody else don’t you?* (P30 Female 77 OEP)
Benefits of the exercise programmes	Pleasure Boredom	*It has got to be enjoyable or you don’t keep it up you know you lose interest so you know I think that… you need plenty of variety in exercise as well otherwise it can become boring*. (P15 Female 72 FaME) *I was pleased when it finished. It wasn’t terribly difficult and I got a bit bored with it*. (P23 Female 81 OEP)
	Social interaction and isolation	*I think that would have encouraged me to sort of oh yes I can do this and I couldn’t do it before, quite interesting if you talk to other people who are doing the programme, I did come across a lady who was doing it, and I chatted to her and we have a bit of a smile and a laugh so even if we were doing it at home, to perhaps have met some other people who were doing it at home* (P01 Female 70 OEP)
	Physical improvement	*I have suffered with back injuries throughout my career and we have added some more different exercises to that programme which helps me with strengthening my back type of thing*. (P27 Male 76 OEP) No difference *I don’t know how much I benefitted, I really don’t…No, no, only that I have done it*. (P09 Female 78 OEP)
	Knowledge and understanding	*I can still remember you know how she told us to get up if we had fallen down*. (P07 Female 72 FaME)
Reactions to the end of the FaME or OEP programmes		*I quite missed because it was Friday mornings, I quite missed that. It did motivate me for a bit to do more walking but then I am busy anyway so… yes I was disappointed, I was hoping that somebody might have carried it on… I would have personally paid for that session to carry on*. (P04 Female 72 FaME)

### Choice in the type of exercise programme

ProAct 65+ participants were randomised to either structured class-based exercise (FaME) or more flexible home-based exercise (OEP). Participants were not able to choose which they received; however, many participants indicated a strong preference for one or other group.

*“Oh I was pleased, I didn’t want to do the home ones at all.”* (P10 Female 88 FaME)

### Commitment, discipline and motivation

Most participants in the FaME classes talked about the classes being a commitment which acted as motivation to exercise and provided a disciplined approach, which the OEP home exercise programme did not. Having to attend a class at a set time ensured that time was set aside for exercise, whereas the OEP home exercise programme participants tried to fit around the activities of everyday life, including caring responsibilities, and which often competed with doing the exercises. FaME participants also spoke about being ‘pushed’ to do exercise by class instructors, whereas OEP participants talked about having to ‘push themselves’ to exercise.

*“I might have slipped up a little bit, and not always done them whereas when it is once a week and you’re joining other people, it is much easier to do exercises with other people than on your own.” (*P08 Female 75 FaME)

*“At home if it was a cold day or I was a bit busy or felt a bit tired I would think oh I will perhaps give it a miss today, you know or … I wouldn’t be so keen to do them yes.”* (P01 Female 70 OEP)

### Benchmarking, feedback and monitoring

FaME participants reported the benefits of being able to compare their exercise capability and/or compete with others in their class. They reported satisfaction of knowing they were ‘doing the exercises correctly’. Some, but not all, OEP participants spoke of the motivational benefits of completing exercise diaries and knowing someone was going to look at them.

*“In fact I tried to beat everybody else all the time.”* (P18 Male 78 FaME)

*“I think … with having the chart, being kept to account made me do the exercises… I wouldn’t have done of my own volition no.”* (P01 Female 70 OEP)

### Benefits of exercise programmes

Four sub-themes emerged about benefits of the exercise programmes. These were pleasure and boredom, social interaction and isolation, physical benefits, and knowledge and understanding.

#### Pleasure and boredom

Most FaME class participants described the classes as enjoyable and spoke about the importance of exercise being enjoyable if it is to be sustainable. Some OEP participants also spoke about enjoying the home exercises.

*“I quite enjoyed it actually, it is a time factor again, I used to find myself erm thinking I have got to do my exercise, do you know what I mean?…but I did enjoy it, I did, I did.”* (P02 Female 80 OEP)

Boredom related to the exercises was given as a reason for stopping and was more commonly mentioned by the OEP participants. FaME participants also described the home exercises that accompanied the FaME classes as boring.

*“Well I tried to do some exercises at home as well because you had to do them in conjunction with the exercise class and I don’t know why but they are not as enjoyable doing them at home on your own.”* (P06 Female 71 FaME)

#### Social interaction and isolation

The social benefits of exercise were important for both the FaME and OEP participants. Meeting people was considered important and enjoyable for the majority of the FaME participants, while OEP participants spoke about missing out on social interaction or feeling isolated.

*“The friendship, the actual exercises, the laughs that we had when people got it wrong, the fun, it was very nice, it was good.”* (P12 Female 84 FaME)

#### Physical improvements

Both FaME and OEP participants described a range of physical improvements that they experienced from taking part in the exercise programmes. These included feeling better, increased flexibility, suppleness, strength, fitness and higher energy levels.

*“I felt better after the exercises…I found myself much more supple in bending down and there is no creaking and things like that.”* (P14 Male 85 FaME)

Participants also expressed that knowing the exercise was ‘doing them good’ or realising that it met their needs, motivated them to continue with it, although not all participants felt that they had benefitted from the exercise programme.

*“I know that it was doing me good because muscles were aching you know and things like that and I thought this is doing me good, and if you think it is doing you good, you will continue won’t you?”* (P02 Female 80 OEP)

#### Knowledge and understanding

Gaining awareness of the different muscles and being able to understand different areas of the body was viewed as beneficial for a number of participants. In addition, participants valued things they had been taught during the exercise programmes which they felt gave them confidence or empowered them.

*“It learnt me to do things that you wouldn’t do…I just tried them out, do them again… I learnt to do it the right way you know walking, or walking and all these different things that we learnt.”* (P25 Female 82 OEP)

#### Reactions to the end of the FaME or OEP programmes

When speaking about the end of the FaME programme, almost all of the FaME participants expressed disappointment about the classes ending and would have welcomed follow-up classes in order to maintain benefits gained.

*“Erm… disappointed. It needs a follow up really if you are wanting to help people getting older because that… it is like taking a step forward and then you just slip back, human nature.”* (P18 Male 78 FaME)

OEP group participants expressed mixed feelings about the programme ending. Some expressed disappointment, particularly relating to the motivating effect of completing the exercise diaries, while many others felt relieved that the programme had ended. For some, the end of the programme was seen as allowing them to return to normal routines and life.

*“A bit disappointed really. I would have liked to have carried on doing it because filling those papers in as well every month gave you the incentive to do it.”* (P30 Female 77 OEP)

*“I can’t really say I felt any… because it was finished and then I would think oh thank god that is done, just stopped it and carried on with my normal everyday activities.”* (P20 Male 77 OEP)

## Discussion

Our analysis identified five key themes that are important when planning the commissioning and provision of strength and balance programmes for older people. We found that it is important for individuals to have choice over the type of exercise they undertake. For some, having regular allocated time with the social interaction offered by group class exercise is a motivator for exercise completion, while for others having the flexibility to exercise in any setting (e.g. at home) and at any time is valued. We found that self-monitoring and feedback is important in encouraging participation, as is enjoying the exercise, seeing physical improvements in oneself and others. Acquiring new knowledge about exercise can be empowering and finally planning programme exit routes is key to enabling exercise continuation and long-term maintenance. Understanding these factors is important if programmes are to be acceptable and for programme attendance and completion to be high.

### Strengths and limitations of the study

Our study reports on the experiences of people that have participated in two types of evidence-based strength and balance training programmes aimed at older people at risk of falling: home-based OEP and group-based FaME programmes. These programmes are referred to in national policy documents in England such as the Falls and Fracture Consensus Statement 2017 (Public Health England, 2017) as appropriate programmes that should be available to people at risk of falling.

We undertook a rigorous approach to the analysis with two researchers coding the data to ensure consistency and we included a lay perspective in the coding to provide a non-academic interpretation of the data. We also used baseline data from the ProAct 65+ trial to ensure that we interviewed a range of people with different levels of baseline physical activity, age, sex and study arm.

As with similar studies of exercise interventions, there may have been some selection bias in terms of which people who agreed to participate. Current and previous activity levels indicated that our interviewees were more physically active than many older people and 21 of the 30 interviewee stated that they engaged in regular weekly exercise, continuing the patterns of exercise undertaken by those in the ProAct 65+ trial. Otherwise, interviewees were broadly representative of those who participated in the ProAct65+ trial as a whole, with a mean minutes of moderate-to-vigorous physical activity per week of 173 for the interviewed FaME group and 177 for the interviewed OEP group versus 171 for the baseline FaME group in ProAct65+ and 193 for the baseline OEP group.

Finally, recalling the intervention was a problem for some participants due to the long period of time between the ProAct65+ trial and the Keeping Active project (between approximately 3 and 7 years). Participants were not always able to recall specific details about the study.

### Comparisons with existing literature

Many of our findings are consistent with other literature on barriers and facilitators to exercise in a range of exercise programmes, including falls-prevention programmes. In their systematic review of reviews, Olanrewaju *et al*. describe three categories of contextually important factors for exercise participation: predisposing factors such as health, motivation, social support and previous exercise experience; enabling factors such as transport, convenience and positive reinforcement and need factors including referral or recommendation to exercise by a health professional (Olanrewaju *et al*., 2016).

While we found themes relating to predisposing factors and enabling factors, the importance of referral or recommendation from a health professional (Dye and Wilcox, 2006, Wilcox *et al*., 2005), did not emerge as a theme in our interviews. We did, however, find themes relating to the importance of motivation (feeling motivated by the instructor, by others or by self-monitoring using diaries all being facilitators), instructor and peer support, and seeing others as role models. Themes relating to fear of falling, feeling fatalistic about aging and inactivity, and health more broadly did not emerge in our study.

Many of the enabling factors were consistent with our study. For example, the issue of convenience of programme setting (e.g. being at home or a set time each week), seeing as the expertise of the instructor as imparting important knowledge, and positive reinforcement all emerged. We did not find evidence that transport (Wilcox *et al*., 2005), or programme charges, communication and information and having safe access were important factors for our participants.

We found that continuation of programmes beyond their scheduled end was important to participants, with many feeling disappointed that the trial ended. Consistent with this, other studies have shown that exit strategies such as structured maintenance programmes or interventions to transition from structured to unstructured programmes are important in ensuring the maintenance of physical activity (Rejeski *et al*., 2009, Duru *et al*., 2010, Voukelatos *et al*., 2015, Luten *et al*., 2016). We also found that participants sometimes found the programmes insufficiently challenging. It is important that strength and balance training is progressive and tailored to individual abilities (Charters, 2013) and our findings support the use of quality assurance or fidelity monitoring mechanisms to maintain programme quality (Skelton *et al*., 2005).

Unlike other studies of falls-prevention exercise uptake, we did not find themes emerging around concerns about body image (Wilcox *et al*., 2003, Mathews *et al*., 2010), cultural or gender barriers (Lewis *et al*., 1997, Snodgrass *et al*., 2005), stigma associated with exercises aimed at older people or falls prevention (Bunn *et al*., 2008), or cost (Bopp *et al*., 2004, Wilcox *et al*., 2005, Horton and Dickinson, 2011, Mathews *et al*., 2010, Smith *et al*., 2012). We found very little evidence that people thought exercise in older age was inappropriate or unachievable (Boehm *et al*., 2013) and little reference to previous exercise experiences and how this influence exercise self-efficacy (Bunn *et al*., 2008).

## Conclusions

Our findings have important implications for commissioners and providers of strength and balance exercise training programmes if uptake is to be maximised by those who will benefit most from them. These include the importance of a wide and varied local offer of strength and balance exercise programmes that suit individual preferences and lead on to sustained access to strength and balance exercise opportunities. The importance of the social benefits of programmes should not be underestimated and in fact should be supported in order to foster peer-led motivation, enjoyment and ultimately maintenance of physical activity. Finally, programmes need to be individually tailored and progressive in order to maintain interest in exercise participation and to achieve maximum health gains. Commissioners should consider ensuring quality mechanisms are in place to be assured of programme progression.
